# Non-interacting proteins may resemble interacting proteins: prevalence and implications

**DOI:** 10.1038/srep40419

**Published:** 2017-01-13

**Authors:** Guillaume Launay, Nicoletta Ceres, Juliette Martin

**Affiliations:** 1 Univ Lyon, CNRS, UMR 5086 MMSB, 7 passage du Vercors F-69367, Lyon, France

## Abstract

The vast majority of proteins do not form functional interactions in physiological conditions. We have considered several sets of protein pairs from *S. cerevisiae* with no functional interaction reported, denoted as non-interacting pairs, and compared their 3D structures to available experimental complexes. We identified some non-interacting pairs with significant structural similarity with experimental complexes, indicating that, even though they do not form functional interactions, they have compatible structures. We estimate that up to 8.7% of non-interacting protein pairs could have compatible structures. This number of interactions exceeds the number of functional interactions (around 0.2% of the total interactions) by a factor 40. Network analysis suggests that the interactions formed by non-interacting pairs with compatible structures could be particularly hazardous to the protein-protein interaction network. From a structural point of view, these interactions display no aberrant structural characteristics, and are even predicted as relatively stable and enriched in potential physical interactors, suggesting a major role of regulation to prevent them.

Proteins do not act alone, but in interaction with other macromolecules: DNA, membranes and other proteins. At the genomic scale, the collection of all protein-protein interactions of an organism, its interactome, can be modeled as a network that provides a framework to understand, interpret, and question the biology of this organism^1^,^2^,^3^. The protein-protein interaction network of the best studied organism in this respect, yeast *Saccharomyces cerevisiae*, is remarkably sparse: about 0.2% of the possible protein-protein interactions actually occur, with an estimation of 37,600 direct interactions between about 6000 proteins^4^.

What about the 99.8% pair interactions that do not take place? How are they prevented in the dense milieu of a living cell? The fate of proteins is indeed regulated at many levels: expression, translation, and localization of proteins are controlled, and post-translational modifications of the proteins modulate their interactions. In addition, the physical interaction between two proteins relies on their structural properties: shape complementarity and favorable atomic contacts *via* charge complementarity, hydrophobic patches, hydrogen bonds and salt bridges^5^,^6^,^7^,^8^,^9^,^10^.

Logically then, 3D structures have been exploited as a source of information for the prediction of protein-protein interactions^11^,^12^,^13^,^14^,^15^,^16^,^17^,^18^,^19^,^20^,^21^,^22^. In the literature, 3D structures are integrated using two different rationales: (i) either one can rely on the (more or less distant) homology between proteins, to infer interaction based on known structures, or (ii) one can rely on the intrinsic structural properties of the proteins, independently of their similarity with known structures. We here briefly review the main approaches dedicated to predict whether or not two proteins interact using these two logic.

Aloy and Russell^11^,^12^ reported the first homology-based method. They used homologous complexes to predict the interaction between candidate proteins and also to derive statistical potentials to score the predicted interaction models. These scores measured the preservation of interface atomic contacts seen in experimental complexes, allowing the distinction between interacting pairs (preserving the contacts) and others. Other groups followed the same direction, with various ways of scoring the models: statistical pairwise potentials combined with sequence identity^23^ or physics-based scoring combined with conservation and template similarity^24^. Multiple threading techniques were also proposed to exploit distant homology relationships^15^,^16^,^17^,^25^; in this case, models are scored by the threading potential, alone or combined with external information such as co-localization and functional annotations^15^,^25^.

In the absence of detectable homology, interaction can be inferred from the comparison with experimental complexes at the structural level, as in the PrePPI algorithm^14^,^26^. In PrePPI, interaction models are scored by the similarity of their interface with the one of the matching structure, in combination with other functional clues (co-expression, functional similarity, phylogenetic profile similarity). Instead of global structure comparison, other methods rely on local structure comparison, restricted to the interfaces^19^,^27^,^28^. Scoring hot spot residue conservation helps to reduce the number of false positives, but these methods still generate a large number of candidate interactions. Let us note that this type of technique, not homology-based *per se*, still relies on homology, since they use interface similarity or conservation measures. In parallel with these explicit comparisons of candidate proteins to known complexes, one machine learning method has been proposed so far, in which the trends of known complexes are used to train a support vector machine classifier^18^: it was shown that structure-based information improves prediction over sequence-only information.

Until now, few methods have tackled the challenge of interaction prediction using intrinsic structural properties, without comparison with available complexes. Different groups used *ab initio* docking to generate interaction models, based solely on the 3D structures of the potential partners^20^,^21^,^22^,^29^. Results are contradictory, but it seems highly difficult to discriminate true from false complexes generated by docking. Indeed, *ab initio* docking algorithms always generate acceptable models, as judged by their scoring functions.

There is no unanimity at the end, on the decisive contribution of 3D structures in the prediction task. A majority of methods use them in the context of homology, and supplement the structural information with other sources of functional information, which are proxies of regulation. So what is the weight of intrinsic structural properties *versus* regulation of protein fate in the existence or absence of protein-protein interactions *in vivo*? To answer this question, we focused on non-interacting proteins, i.e., protein pairs for which no functional interaction is reported, and their 3D structures, and we quantified how frequently they can be mistaken for interacting proteins, by comparison with experimental structures.

Using global structure comparison, we found that up to 8.7% of non-interacting protein pairs in *S cerevisiae* might be similar to interacting pairs, a deluge of potential interactions in comparison with the less than 1% of known functional ones. The non-interacting pairs that are similar to experimental complexes turn out to be significant destabilizers of the native protein-protein interaction network, being more central than other non-interacting pairs. Interestingly, we found no intrinsic structural determinant that could distinguish between interacting and non-interacting pairs, even with a sophisticated physics-based method of binding affinity prediction. Two orthogonal approaches, based on threading scores and interologue detection, allow us to estimate that 9 to 50% of non-interacting pairs that are similar to interacting pairs could interact *in vitro*. On the practical side, we conclude that current tools cannot distinguish low-resolution models of interacting from non-interacting protein pairs. On the theoretical side, we propose that this lack of distinction reflects a real resemblance between interacting and some non-interacting pairs, implying a major role of biological regulation to repress deleterious interactions between proteins with compatible structures.

## Results

### General Protocol

**Figure 1:**
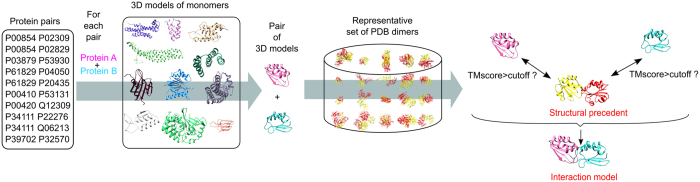
Structural comparison protocol used in this study. Each pair of proteins with available 3D models is submitted to structural comparison with binary complexes from the PDB. When both 3D models are similar to constituents of a PDB complex, the PDB complex is termed the *structural precedent* of interaction. The model derived from the structural matching is termed the *interaction model*.

In this study, we compare the 3D structures of non-interacting protein pairs of *S. cerevisiae* to a set of experimental dimers from the PDB^30^. The general protocol is summarized in Fig. 1. The comparison is made on monomers. In case where both monomers of a pair display significant structural similarity with an experimental dimer, this dimer is termed a “*structural precedent”.* Protein interactions happen through interfaces with specific properties, which are not taken into account by the global structural comparison. Additional criteria are thus used to ensure the existence of a potential interface between monomers (see Methods). When a structural precedent is found, it can be used to derive an “*interaction model*,” by superimposition of the monomers onto the structural precedent. We use the interaction models thus formed to perform additional tests on the interface properties, namely conventional structural features, affinity prediction and contact conservation.

Here, we perform the structural comparison in order to evaluate the extent of the similarity between interacting and non-interacting protein pairs and to assess the weight of the structural information in the prediction task. We do not imply that the existence of a structural precedent indicates an interaction. It only indicates a global structural compatibility between proteins.

To evaluate the current coverage of the protein-protein interaction network by the set of experimental dimers, we did the same comparison using datasets of reported interactions. Note that, in predictive studies, it is customary to remove homologous proteins from the reference set in order to test the performance of the method^31^. Here, our goal is not to predict the structure of the complexes or the interaction between proteins, but rather to estimate the coverage of the experimental data. It is thus important to keep every protein; otherwise we would underestimate the real coverage of the reference dataset.

### Selection of Datasets of Non-Interacting and Interacting Proteins

There is no consensus yet on a gold standard for non-interacting datasets in the literature, but rather several alternatives based on different selection strategies^32^,^33^,^34^,^35^,^36^,^37^,^38^. Here, we consider five different non-interacting datasets:

1. A random sampling among unobserved interactions^32^,
2. A balanced sampling among unobserved interactions, which takes into account the number of connections of the proteins in the native network^32^,
3. Proteins with different subcellular localization,
4. Pairs with the most dissimilar functional annotations^35^,
5. Pairs selected after two-hybrid data post-processing, in order to reduce the false negative rate^33^.

For the interacting proteins, we considered four different datasets: one set of high confidence physical interactions (positive BRS) from BioGrid^39^, one set restricted to direct interactions (positive BRS-direct), another set of direct interactions (positive KUPS) from KUPS^35^, and the Ito core data set^40^ (positive Ito).

### Coverage of Structural Information

For each pair of proteins from our different interaction data sets, 3D models of monomers are used as input to identify structural precedents in the reference set of experimental dimers. The identification is thus contingent on the availability of 3D models. The fraction of proteins with available 3D models is similar across all the data sets: around 76% for single proteins and 60% for protein pairs (see Table 1). Two datasets display a lower pair rate, around 53%: the “*positive KUPS”*, and the “*negative GO”* datasets. Note that they were retrieved from the same resource^35^.

### Identification of Structural Precedents for Interacting and Non-Interacting Pairs

**Figure 2:**
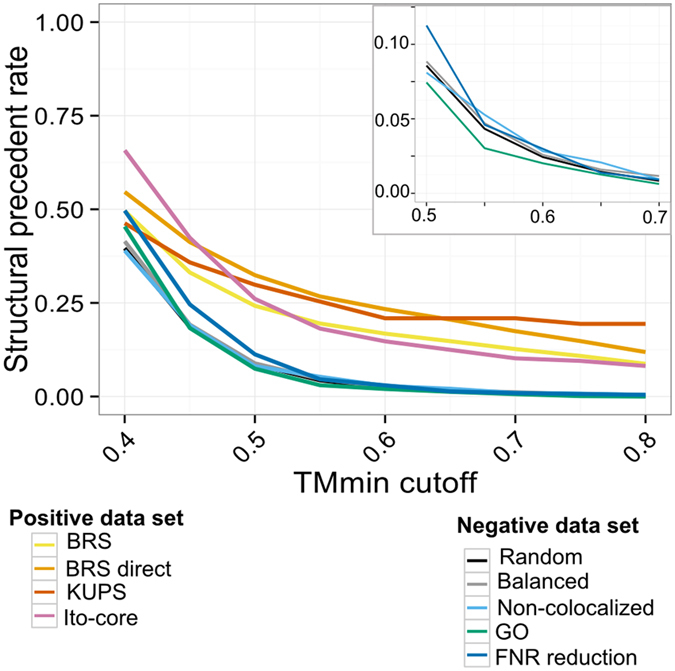
Ratio of pairs with structural precedents in each dataset. The right inset is an enlargement over the region around TMmin cut-off = 0.6 for negative datasets.

The structural similarity between 3D models and experimental structures is assessed by the TM-score^41^, ranging from 0 (no similarity) to 1 (identical structures). The minimum TM-score, TMmin, summarizes the similarity between a candidate pair and an experimental structure^42^. In Fig. 2, we show the fraction of protein pairs with structural precedents, with respect to the number of pairs with 3D models, as a function of the TMmin threshold.

The threshold value has a clear impact on the rate of detection of structural precedents. Initially, the value of 0.5 was proposed to detect proteins with identical fold^41^. In another study, a value of 0.4 was used to predict interaction modes, since it is the observed limit between complexes with similar and different binding modes^42^,^43^. This choice was challenged in a subsequent study, which suggested a value of 0.6 to produce acceptable models^31^.

Here, a threshold of 0.4 yields rates of structural precedents in overlapping ranges for non-interacting and interacting proteins: 40% to 50% for non-interacting pairs and 45 to 65% for interacting pairs. This means that roughly half of the protein pairs are similar to a known experimental dimer, even when the proteins do not interact. As expected, higher thresholds lead to a decrease in the rate of structural precedents, but also to a clear distinction between non-interacting and interacting pairs. At TMmin threshold equal to 0.6, the rate of structural precedent ranges from 15% to 23% for interacting pairs, *versus* 2% to 3% for non-interacting pairs. In the rest of the study we will use the 0.6 threshold.

Interestingly, the rate of structural precedents is more variable across the datasets of interacting pairs than across the non-interacting pairs. A possible factor is the disparity of experimental techniques. The “*positive BRS direct*” and the “*positive KUPS*” datasets both contain only direct interactions and they have consistent rates of structural precedents (23 and 21%). By contrast, the “*positive BRS”* dataset, which contains direct and indirect interactions, has a lower rate of 17% of structural precedents. However, this factor does not explain why the “*positive Ito”* dataset, obtained by large-scale yeast-two hybrid, has a lower rate of 15%. This illustrates the recurrent problem of the interaction data quality^44^. Notably, the strategy used to select the non-interacting pairs has very little impact on the rate of structural precedents (see Table S1).

### Extrapolation of the Rate of Structural Precedents of Non-Interacting Pairs

We now focus on the numbers obtained at the 0.6 threshold and what they mean. One could object that the low rates of structural precedents in the non-interacting datasets are merely noise due to false negatives (actual interactions not reported as such). But let us recall that the ratio between effective and potential interactions in yeast is about 0.2% (37,600 physical interactions, among 6000 proteins^4^). Here, we observe 15 to 23% of structural precedents for interacting pairs. If all our non-interacting datasets were contaminated by false negatives, in a purely random manner, we would then expect [15–23]%*0.2% = [0.03–0.05]% of structural precedents. Instead of 0.03–0.05%, we obtained 2–3%, i.e. 40 to 100 times more than expected by chance (Fisher test p-value = 3.7e − 3, see Table S2). This can mean that (1) either non-interacting protein pairs also have structural precedents or (2) all the non-interacting datasets are richer in real interactions than expected by chance (by a factor 40 to 100 times). This is extremely unlikely, since we considered five non-interacting datasets, generated by different and orthogonal approaches.

The rates of structural precedents are high if we consider the total pool of potential interactions (99.8% non-interacting, 0.2% interacting), and even more if we take into account the incompleteness of the PDB. Indeed, using the rate of structural precedents of interacting pairs as a proxy of the PDB completeness (23% in the most optimistic scenario) and the lowest observed rate of structural precedents (2% at least), we obtain 2/23*100 = 8.7% of non-interacting pairs with structural precedents. This is the expected rate if the PDB were complete in terms of interactions (i.e. 100% structural precedents for interacting pairs). Of course, this estimation is based on our observations for available 3D models, i.e. roughly 60% of interactions. In addition, in doing so, we postulate that the growth of the PDB from 23 to 100% is equally affecting the rate of structural precedents for interacting and non-interacting pairs. The PDB is not an unbiased sample of the protein structural space and one could object that the new structures might be more likely to match the interacting pairs than the non-interacting ones. If that were the case, our current estimation would indeed underestimate the rate of structural precedents of non-interacting pairs.

### Potential Impact of Non-interacting Pairs with Structural Precedent on the Interaction Network

**Figure 3:**
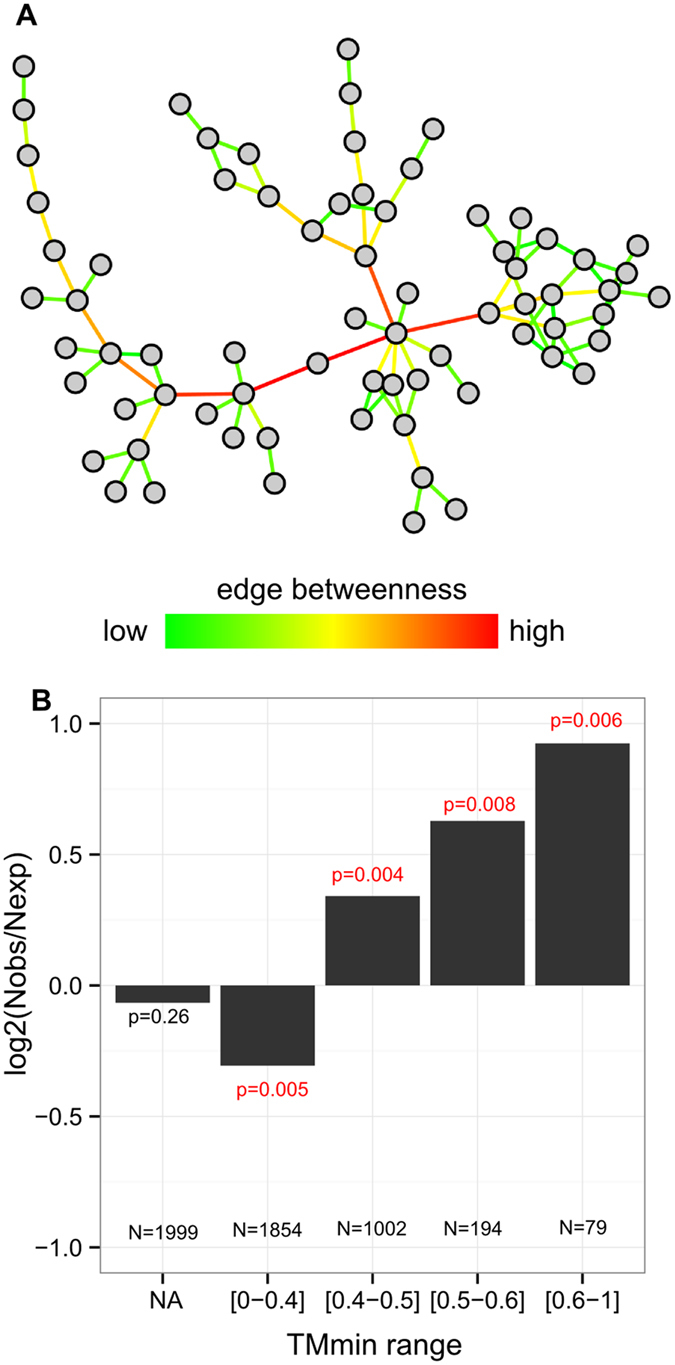
Network analysis of non-interacting pairs. (**A**) Illustration of the edge betweenness measure. Figure generated by Cytoscape^64^. (**B**) enrichment in bottlenecks according to the TMmin value, when extra interactions are added to the native network. The number of interactions and the p-value of the Chi-squared residuals are reported in each case. NA means that the non-interacting pairs were not submitted to structural comparison due to the lack of models.

Here, we have found that a substantial fraction of non-interacting protein pairs indeed resemble interacting proteins. What would be the biological impact of these extra interactions if they were to occur? In particular are they more dangerous than other interactions? To answer these questions, we analyzed the localization of the non-interacting pairs in the native protein-protein interaction network. The structure of the network is here used as a proxy of the biological system integrity. Each non-interacting pair was added separately to the native network, and the centrality of the extra edge was measured by its betweenness value, which is the count of the shortest paths in the graph that go through that edge^1^,^45^, as illustrated in Fig. 3A. The bottleneck edges are the top 10% with the highest betweenness. Because bottleneck edges are key connections in the network, they are critical in terms of the perturbation of the network topology. Is has been shown, in physiological context, that bottlenecks are linked to gene essentiality^45^.

As a physiological network, we considered the network formed by the 5621 interactions from the “*positive BRS*” dataset. Protein-protein interaction networks typically contain permanent but also transient interactions that are difficult to detect. In our case, the ‘positive BRS’ dataset gathers interactions detected by several methods. Notably, one third of the 5621 interactions are detected by yeast-two hybrid, which is well-suited to detect transient interactions^44^. Unconnected components were removed, leaving a subnetwork formed by 5374 interactions between 1934 proteins. We introduced extra interactions from the *“negative balanced”* dataset, restricted to the 5128 interactions between the proteins present in the native network. The enrichment in bottleneck edges is expressed by the log-odd score of the observed *versus* expected number of bottlenecks in each category. As shown in Fig. 3B, we observed a significant enrichment of bottlenecks in non-interacting pairs with structural precedents at high TMmin: log-odd = 0.9 at TMmin > 0.6 (18% of bottlenecks). This enrichment persists at medium TMmin: log-odd = 0.6 at TMmin [0.5–0.6] (16% bottlenecks), log-odd = 0.4 at TMmin [0.4–0.5] (12.5% bottlenecks). Such enrichment is not observed in the native unperturbed network (see Figure S1).

Our results thus show that non-interacting pairs with structural precedents correspond to key extra connections in the network. They are more central than other non-interacting pairs, meaning that they create more shortcuts when added to the native network. This suggests that they would have more dramatic biological consequences.

We also analyzed the functional annotations of the proteins involved in pairs with high TM scores (>0.6). The following annotations were over-represented for non-interacting pairs but not for interacting pairs: “biological regulation” (p-value = 4.4e − 2), “regulation of cellular process” (p-value = 4.4e − 2), “signal transduction” (p-value = 4.4e − 2), “intracellular signaling pathway” (p-value = 4.4e − 2), “signal transmission” (p-value = 4.4e − 2), “regulation of biological process” (p-value = 4.4e − 2), “signaling process” (p-value = 4.4e − 2), “signaling pathway” (p-value = 4.7e − 2) and “signaling” (p-value = 4.7e − 2). These pairs are thus enriched in signaling proteins.

### Analysis of Protein Abundance

We analyzed the abundance of the proteins involved in non-interacting and interacting pairs, on the dataset used for the network analysis. We found that negative pairs are slightly but significantly depleted in highly abundant proteins compared to positive pairs (see Figure S2). However, negative pairs with structural precedents (TMmin > 0.6) are not significantly different from other negative pairs. We thus see no evidence of a particular down-regulation of non-functional interactions acting on negative pairs with structural precedents, *via* low protein abundance. Let us note that the control of protein abundance is one mechanism of control among several others. The global regulation results from the juxtaposition of these different mechanisms. The relative contribution of each mechanism is unknown, and probably difficult to assess. It is likely that protein abundance alone has a mild effect, as suggested by the moderate correlation between protein abundance and propensity for promiscuous interactions found by others^46^. This might explain why we did not find a significant signal in protein abundance.

### Positive and Negative Models Are Not Separable Using Simple Structural Descriptors

**Figure 4:**
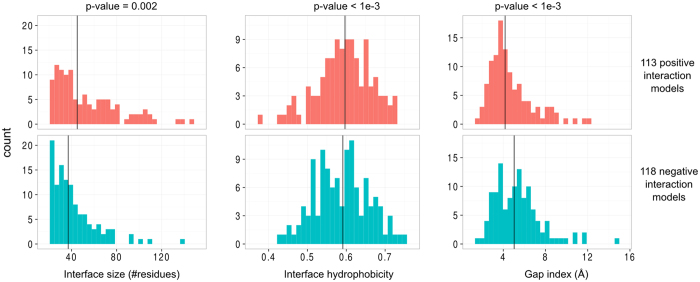
Characterization of interaction models in the TM range 0.6–0.8 using conventional interface descriptors. In each panel, vertical lines represent the median of the distribution. The p-value of the Cramer test comparing the two distributions is shown for each descriptor.

Having shown that a considerable fraction of non-interacting protein pairs are similar to interacting ones, and are potentially highly deleterious, we now analyze the features of the hypothetical interaction models to which they correspond. More precisely, we compare the interaction models, obtained by superimposition of the 3D models onto the PDB dimers, for the interacting pairs—termed positive models—and non-interacting pairs—termed negative models. In order to work with models of comparable confidence, we selected interaction models with TMmin in the range [0.6–0.8]: 113 models of interacting pairs from the *“BRS direct”* and *“KUPS”* datasets, and 118 models of non-interacting pairs from the union of all negative datasets (see Figure S3). On these pairs, we analyzed (i) features of the interaction models: interface size, interface hydrophobicity, gap index, and (ii) features of the structural precedents: classification as homo or hetero-dimers, obligate or non-obligate complexes and dimers *versus* parts of higher order complexes. Concerning the interaction models, none of these features could discriminate between positive and negative models: although statistically different, the distributions are very similar, see Fig. 4. Negative models cannot be excluded because they have tiny or poorly packed interfaces, for example. In the same way, we did not find any strong disequilibrium between positive and negative pairs in terms of structural precedents classification (see Figure S4). These observations suggest that positive and negative models are difficult to separate using simple intrinsic structural descriptors.

### Binding Affinity and Contact Conservation of Interaction Models

**Figure 5:**
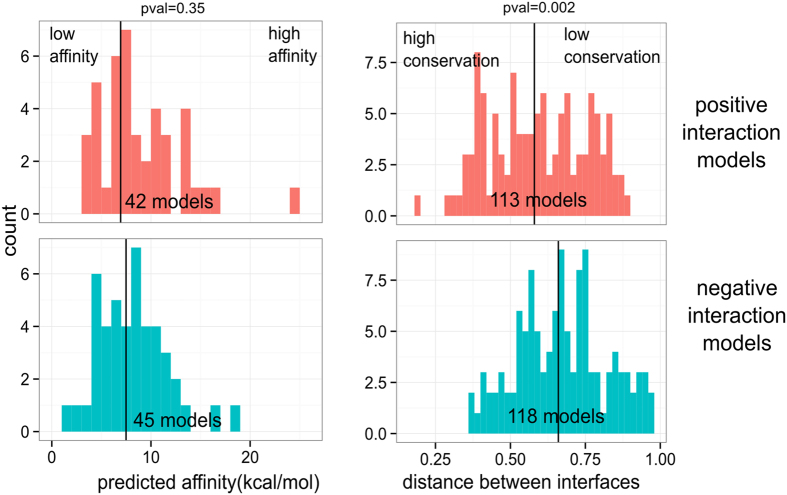
Characterization of interaction models in the TM range 0.6–0.8 using predicted binding affinities and distance between interface signatures. In each panel, vertical lines represent the median of the distribution. The p-value of the Cramer test comparing the two distributions is shown for each descriptor.

We also tested more sophisticated tools to describe interaction models: binding affinity prediction and distance between interface signatures (see Figure S5). Binding affinities are predicted using the PaLaCe coarse-grain model^47^. Here, it is important to emphasize that the interaction models result from the superimposition onto the PDB dimers; they are not refined, except for the removal of steric clashes, before affinity prediction. They are thus probably of moderate quality and source of noise in our observations. First, let us note that the proportion of models predicted as stable is similar for interacting and non-interacting pairs (see Figure S6). As seen in Fig. 5, models of interacting pairs marginally populate the high-binding affinity region, compared to non-interacting pairs. The difference between the two distributions is not statistically significant (p-value = 0.35). Thus even with sophisticated energetic models, non-interacting models appear as plausible as interacting models.

Lastly, we computed the distance between interaction models and their structural precedents, based on the composition of the pairwise contacts at their interface, see Fig. 5. This distance is indeed an implicit way to capture the homology relationship between complexes. Although both distributions lie in the same range, there is a visible bias toward low distances for interacting pairs, indicative of higher contact similarity. Some models of interacting pairs are thus closer to experimental structures than the negative ones. Even if we did not rely on sequence comparison, it seems that we still retrieved cases of homologous complexes. We thus confirm the utility of homology-based measures to reveal evolutionary traces. These results are in good agreement with the fact that structural features need to be used in combination with non-structural features in current predictive methods^14^.

### A Proposed Hypothesis

We thus found a non-negligible quantity of non-interacting pairs similar to interacting pairs in the interactome of *S. cerevisiae*. By extrapolation, we estimate the prevalence of these non-interacting pairs around 8.7%. On the one hand, their network centrality indicates that the corresponding interactions would have a particularly deleterious effect on the physiology of the cell, if they were to occur. On the other hand, despite their notable dangerous nature, they display no aberrant structural features, and are even predicted to be relatively stable. How to reconcile these two observations?

The concept of misinteraction avoidance could help to explain this apparent discrepancy. Misinteraction avoidance denotes the natural selection against non-functional interactions. This phenomenom has been suggested to act as an evolutionary force, decreasing the evolution rate of highly expressed proteins^48^, and biasing their surface composition^46^.

We propose the following hypothesis: since non-functional interactions between proteins with compatible structures correspond to critical shortcuts in the network, they must be tightly down-regulated. In the most extreme scenario, the misinteraction avoidance will result in a spatio-temporal segregation of these proteins. If these proteins never encounter one another thanks to regulation, there is no driving force to prevent a physical compatibility between them (negative pleiotropy^49^). In other words, they did not evolve to prevent the formation of a complex, because they did not need to. This could explain why interacting and non-interacting pairs with structural precedence are not easily distinguishable using intrinsic features.

### Estimation of the Fraction of Potential Physical Interactors among the Negative Pairs with Structural Precedent

Our hypothesis is that the lack of discrimination between interacting proteins and non-interacting proteins with support is due, at least in part, to the absence of pressure against misinteraction avoidance on the proteins, due to physiological regulation. If this holds true, then some of the non-interacting proteins with structural precedence should be able to interact *in vitro*, as suggested by our affinity prediction results. In support to this hypothesis, we here provide an estimate of the fraction of negative pairs with structural precedent that are potential *in vitro* interactors. To do so, we used two different approaches. One is based on the prediction of interactions by Struct2Net^15^,^16^ and the other one is based on the presence of interologues in the public interaction databases.

The prediction of interactions provided by Struct2Net relies on interfacial energy and alignment scores, obtained by threading sequences onto a library of structural templates. We submitted three different data sets to the prediction: 113 pairs of interacting proteins, 118 pairs of non-interacting proteins with structural precedent in the TMmin range [0.6–0.8] (pairs presented in Fig. 4), and 2018 non-interacting pairs for which no structural precedent could be identified, although both protein models were available. The results of the Struct2Net prediction are shown in Table 2.

As shown in Table 2, 4% of the non-interacting pairs with structural precedents are predicted to interact by Struct2Net. By contrast, less than 1% of non-interacting pairs without precedent are predicted to interact. For interacting pairs, the fraction predicted by Struct2Net is 44%, indicating that the corrected fraction of non-interacting proteins with favorable interfacial energy would be around 9%. This result allows us to provide a first estimate of 9% of the negative pairs with compatible structures that could interact *in vitro*.

In addition, we screened the interaction databases to identify interologues, i.e., homologue proteins with reported interactions. Results are shown in Table 2. Using the protocol described in Material and Methods, we found that 36% of non-interacting pairs with structural precedents have interologues, *versus* 18% only for non-interacting pairs without support. For interacting pairs, the ratio is 70%, yielding a corrected ratio of about 50% for negative pairs with support. It is interesting to note that both approaches show a significant enrichment in the set of non-interacting pairs with support compared to pairs without support.

This last analysis allows us to propose an estimate of 9 to 50% of the non-interacting pairs with precedents that could interact *in vitro*.

## Conclusion

To conclude, our work provides an estimate of the prevalence of non-interacting protein pairs with structural precedents. These pairs of proteins are quite central in the interactome and enriched in signaling proteins. Although potentially hazardous, these pairs give rise to plausible structural models, hardly distinguishable from models of interacting proteins based on intrinsic structural features. A fraction of these pairs (9–50%) are predicted as potential physical interactors by threading scores and network analysis.

This estimate should, of course, be considered with caution, as any estimate obtained by indirect, predictive methods. The ultimate confirmation of physical interaction would require the use of experimental methods like isothermal titration calorimetry (ITC). The most probable scenario is that only a fraction of the potential physical interactors identified here would indeed produce a measurable interaction using ITC. However, we want to emphasize that, more important than the estimate fraction itself, we observed an enrichment of potential physical interactors in non-interacting pairs with structural support compared to non-interacting pairs without support. Altogether, our findings support the view of protein-protein interaction as a property to repress.

## Material and Methods

### Interaction Datasets

All the data used in this study are for the model organism *Saccharomyces cerevisiae*. The different datasets of interacting and non-interacting protein pairs are presented in Table 1 and described hereafter. When necessary, the mapping between Uniprot and gene ids was done using the Uniprot REST interface http://www.uniprot.org/uniprot.

Three data sets from the Yu *et al*. study^32^ were retrieved from http://fbs3pcu112.leeds.ac.uk/BRS-nonint/PPI_RandomBalance.html:

- **Positive BRS**: high confidence physical interactions, obtained after filtering out the “HC-BIOGRID-2.0.31.tab” dataset to remove proteins without PFAM domains. The experimental methods supporting each interaction were retrieved from BIOGRID.
- **Negative random:** random sampling of non-interacting pairs within the “positive BRS” list.
- **Negative balanced:** sampling of non-interacting pairs within the “positive BRS” list, while preserving the number of connections of each gene.
- We derived two additional datasets:
- **Positive BRS-direct:** by filtering the “Positive BRS” list using BioGrid data^39^ to select only interactions described by one of the following terms: “Two-hybrid”, “PCA”, “Biochemical Activity”, “Co-crystal Structure”, “Reconstituted Complex”, “Far Western”, “FRET”,
- **Negative non-colocalized:** by filtering the “Negative random” list using Uniprot annotations^50^ to select pairs of proteins with different subcellular localization. Proteins with several localization were removed.

Two datasets were retrieved from the KUPS database^35^ (http://www.ittc.ku.edu/chenlab/kups/), using the following input parameters: size of the data set = 1500, species = Saccharomyces cerevisiae, for the positive set: interactions = direct interaction, detection methods = all, for the negative set: interactions = all, detection methods = all, restricted = yes, selection strategy = functionally dissimilar pairs:

- **Positive KUPS:** by removing self-interactions from the resulting positive list.
- **Negative GO:** by removing self-interactions from the resulting negative list. These pairs are chosen in order to minimize functional similarity between proteins using Gene Ontology annotations (GO)^51^.
- Two data sets were derived from the study by Trabuco *et al*.^33^:
- **Positive Ito-core:** we retrieved the interaction data described in ref. 40 from the IntAct database^52^ (http://www.ebi.ac.uk/intact/). Only interactions annotated as “core” (high confidence pairs with interaction observed at least three times in the experiment) were retained; self-interactions were removed.
- **Negative FNR reduction:** we retrieved an initial list of 214,696 pairs constructed by Trabuco *et al*.^33^ from http://www.russelllab.org/negatives/. This list contains interactions obtained after post-processing of the Ito core data set, in order to reduce the false negative rate (FNR). Interactions in this set were likely tested (single proteins are valid prey and bait). As described in ref. 33, we selected only protein pairs with a shortest path greater than 10 in the protein-protein network, and picked a random sample of these interactions.

### Abundance Data

Protein abundance values were retrieved from the PaxDb4 database (http://pax-db.org/). We considered the ‘integrated’ values, which are the weighted averages over several dataset. Thanks to abundance values, we defined three abundance categories: ‘low abundance’ for proteins annotated as *bottom 25%* or less by PaxDb, ‘high abundance’ for proteins annotated as *top 25*% or more, ‘medium abundance’ for the others. We then defined abundance for a pair of proteins as the lowest abundance in the pair. The rationale is that the low abundance of one protein is a limiting factor for the formation of an interaction.

### Homology 3D Models

Pre-computed homology 3D models of yeast ORFs were retrieved from ModBase (modbase.compbio.ucsf.edu). Some ORFs have several 3D models. Models covering less than 40% of the full length of the target proteins were excluded.

### Representative Set of PDB Dimers

We derived a non-redundant list of 12,379 PDB dimers from the InterEvol resource^53^ http://biodev.cea.fr/interevol/ by filtering out the initial list (INTER70_REFINFO.table: 17,658 dimers, non-redundant at the 70% level, with more than 10 residue contacts at a 5 Å distance threshold) to remove crystallographic interfaces. Some of these dimers are parts of higher order complexes; all are classified as homo/heterodimers and as obligate/non-obligate complexes by the InterEvol resource^53^.

### Structural Comparison

The similarity between 3D models of yeast proteins and PDB dimers was measured using the TM-score, which ranges from 0 (no similarity) to 1 (perfect similarity) using TM-align^41^. For a given protein pair A/B and a given PDB dimer X/Y, the similarity is measured by TMmin, the minimum TM score obtained for A *versus* X and B *versus* Y (and also B/A *versus* X/Y). In case of sufficient similarity (TMmin higher than a given cut-off), the PDB dimer X/Y is termed the *structural precedent* of the pair. The *interaction model* produced by superimposition of the yeast models onto the structural precedent must have an interface of at least 20 residues (at the 5 Å cutoff) and less than three clashes between Cαs. If several 3D models are available for a candidate protein, we considered every possible pair of models, ranked them by decreasing TMmin value, and retained the first case with a valid interface.

### Network Analysis

We used the igraph R package^54^ to select the largest connected component in a network and to compute the edge betweenness.

### GO Annotation Analysis

The over-representation of functional annotations for a set of proteins was assessed using the BINGO plugin for Cytoscape^55^, with the following parameters: GO biological process annotations, hypergeometric test, Benjamini Hochberg correction for multiple testing, threshold for significance = 0.05, use the network as reference set. The over-representation was assessed for pairs with TMmin greater than 0.6.

### Structural Features of Complexes

Conventional structural descriptors of protein-protein interfaces were computed for the interaction models: interface size (number of residues below a 5Å distance threshold), interface hydrophobicity (fraction of interface accessible surface area contributed by hydrophobic residues, obtained with NACCESS^56^), gap volume at the interface measured by SURFNET^57^ and gap index (ratio of gap volume over interface accessible surface area). The Cramer test implemented in the Cramer package^58^ is used to test the equality between resulting distributions for positive and negative pairs. This non-parametric test is well suited to handle non-normal distributions with ties.

### Predicting Binding Affinity of Interaction Models

The binding affinity of the interaction models was predicted with the PaLaCe coarse-grained model^47^. PaLaCe uses a two-tiers protein representation: one or two pseudoatoms per side-chain and an atomistic representation of the main chain. The total energy of a system is given by the linear combination of the unweighted bonded and non-bonded energy terms that constitute the PaLaCe force field (bonds, valence and torsion angles, electrostatics and Lennard-Jones contributions for side-chain pair interactions and backbone hydrogen bonds, and a one-body solvation term that partially compensates for the lack of explicit water). Interactions models were rigid-body minimized to remove steric clashes and only models whose minimization reached convergence (gradient < 10E-05) were kept for affinity prediction. Palace predictions were calculated by subtracting the total energy of each complex (E_AB_) from the total energy of the individual partners in their bound conformations (E_A_ + E_B_), and scaled as follows:


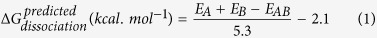


This correction is based on the high correlation (correlation coefficient equal to 0.8) between a representative subset of experimental binding affinities^59^ and the corresponding PaLaCe predictions (data not shown). Models that ended their minimization far away from the initial configuration (rmsd of the shorter chain greater than 5 Å after superimposition of the longer chain) and models that were predicted as less stable than the isolated monomers (∆G < 0) were rejected from the analysis.

### Distance Between Interfaces

Interfaces were compared using the following distance:


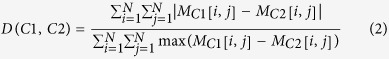


where N denotes the number of amino-acid classes and M_C1_ and M_C2_ are NxN matrices counting the number of interface contacts (less than 12 Å between Cα) in complexes C1 and C2. We tested various amino-acid groupings and opted for N = 7 groups: {V, I, M, C, L}, {A, S, P, T}, {G}, {Y, F, W}, {K, R, H}, {D, E}, {N, Q}. D(C1, C2) is equal to 0 when the interfaces are identical in terms of amino-acid contact types and 1 when they have no contact type in common.

### Prediction of Interactions by Threading scores using Struct2Net

Pre-computed predictions of interactions for yeast proteins by the Struct2Net algorithm^15^ were retrieved from http://groups.csail.mit.edu/cb/struct2net/webserver/. The Struct2Net approach relies on structure-based threading and is independent of functional genomic information such as co-expression or cellular localization.

### Search for Interologues in Interaction Databases

Interologues for protein pairs were retrieved with the following protocol.

In a first step, for each protein sequence of the dataset, a maximum of 500 homologues were extracted from the uniprot database (release 2016_08) using the blastpgp software^60^ (ncbi-blast package 2.2.26) with the default parameters standard (e-value = 0.002, max number of iterations = 3). Hence, each protein of the dataset is associated with a list of homologous proteins.

In the second step, protein-protein interaction data for all the proteins (queries and homologs) were obtained from the Intact^52^, MINT^61^, DIP^62^ (psicquic protocol^63^) and BioGRID^39^ (specific REST interface) databases. Only experimental evidences obtained by the yeast-two hybrid method were considered. Experimental evidence of interaction between the homologs of a protein pair (interologues) indicates a potential homology support for their interaction and is here taken as an indicator of potential physical interaction.

## Additional Information

**How to cite this article**: Launay, G. *et al*. Non-interacting proteins may resemble interacting proteins: prevalence and implications. *Sci. Rep.* **7**, 40419; doi: 10.1038/srep40419 (2017).

**Publisher's note:** Springer Nature remains neutral with regard to jurisdictional claims in published maps and institutional affiliations.

## Supplementary Material

Supplementary Dataset 1

## Figures and Tables

**Table 1:** The data sets of protein-protein interaction used in this study.

Dataset name	Source	Dataset type	Nb proteins	Nb proteins with 3D models	Nb pairs	Nb pairs with 3D models (%)
Positive BRS	Yu *et al*.^32^	Interacting	2245	1714 (76%)	5621	3467 (62%)
Negative random	Yu *et al*.^32^	Non-interacting	2245	1714 (76%)	5621	3326 (59%)
Negative balanced	Yu *et al*.^32^	Non-interacting	2245	1714 (76%)	5621	3445 (61%)
Positive BRS-direct	Yu *et al*.^32^, BioGrid^39^	Interacting	1870	1413 (76%)	2923	1773 (61%)
Negative non-colocalized	Yu *et al*.^32^, Uniprot^50^	Non-interacting	868	655 (75%)	909	531 (58%)
Positive KUPS	KUPS^35^	Interacting	156	117 (76%)	126	67 (53%)
Negative GO	KUPS^35^	Non-interacting	1112	753 (75%)	1498	793 (53%)
Positive Ito-core	Trabuco *et al*.^33^, IntAct^65^	Interacting	783	595 (76%)	750	441 (59%)
Negative FNR reduction	Trabuco^33^	Non-interacting	792	497 (76%)	749	435 (58%)

**Table 2:** Predictions by Struct2Net and search for interologues.

Data set	Nb pairs	Nb predicted as interacting by Struct2Net (%)	Nb with interologues (%)
Interacting pairs	113	50 (44%)	79^a^ (70%)
Non-interacting pairs with precedent TMmin [0.6–0.8]	118	5 (4%)	43 (36%)
Non-interacting pairs without precedent	2018 125^b^	19 (0.9%)—	NA 23^b^ (18%)

^a^Query proteins are excluded from the interologue sets.

^b^The search was done on a random subset of 125 pairs.

NA: non available.
