# Supplementary material for: Non-interacting proteins may resemble interacting proteins: prevalence and implications
Source: Sci Rep. 2017 Jan 13;7:40419. doi: 10.1038/srep40419 (PMC5289270; doi:10.1038/srep40419)
Supplement: Supplementary Dataset 1 [file srep40419-s1.doc]

## Title

Non-interacting proteins may resemble interacting proteins: prevalence and implications

## Authors

Guillaume Launay^1^, Nicoletta Ceres^1^, Juliette Martin^1^

## Affiliations

1: Univ Lyon, CNRS, UMR 5086 MMSB, 7 passage du Vercors F-69367, Lyon, France

| Dataset name | Nb pairs with 3D models | Nb pairs with structural precedent at TMmin>0.6 (%) |
| --- | --- | --- |
| Positive BRS | 3467 | 583 (16.8%) |
| Positive BRS-direct | 1773 | 415 (23.4%) |
| Positive KUPS | 67 | 14 (20.9%) |
| Positive Ito-core | 441 | 65 (14.7%) |
| Negative random | 3326 | 81 (2.4%) |
| Negative balanced | 3445 | 89 (2.6%) |
| Negative non-colocalized | 531 | 15 (2.8%) |
| Negative GO | 793 | 16 (2.1%) |
| Negative FNR reduction | 435 | 13 (3.0%) |

Table S1: Number of pairs with structural precedents TMmin>0.6 cut-off

| Data | Fisher test |
| --- | --- |
| # frequency matrix  # 0.05 % versus 2%  > M       [,1]  [,2]  [1,] 0.05 99.95  [2,] 2.00 98.00  # count matrix, based on the smallest negative dataset: 435 pairs  # before rounding  > C  [,1] [,2]  [1,] 0.2175 434.7825  [2,] 8.7000 426.3000  # after rounding  > C_round  [,1] [,2]  [1,] 0 435  [2,] 9 426 | > fisher.test(C_round)  **p-value = 0.003746** |

Table S2: Fisher test on the rate of structural precedents


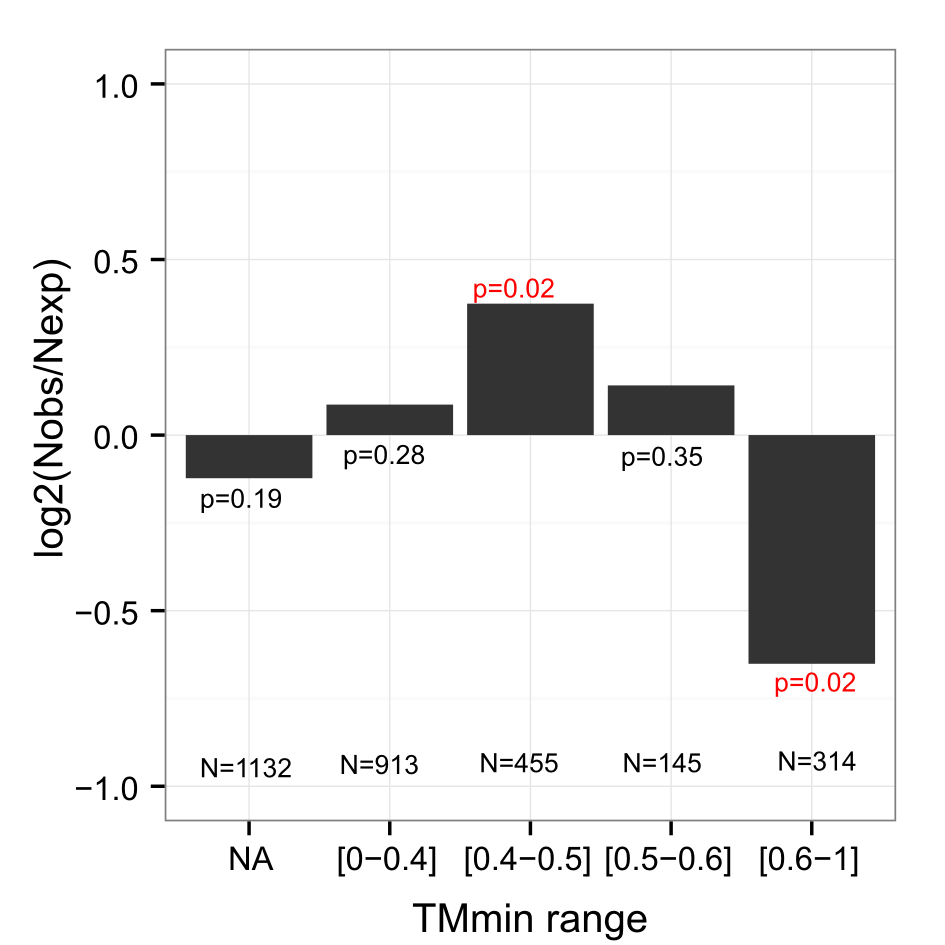


Figure S1: enrichment in bottlenecks according to the TMmin value in the native network. The number of interactions and the p-value of the Chi-squared residual are reported in each case. NA means that the pairs were not submitted to structural comparison due to the lack of models. The enrichment in bottleneck edges is expressed by the log-odd score of the observed *versus* expected number of bottlenecks in each category.


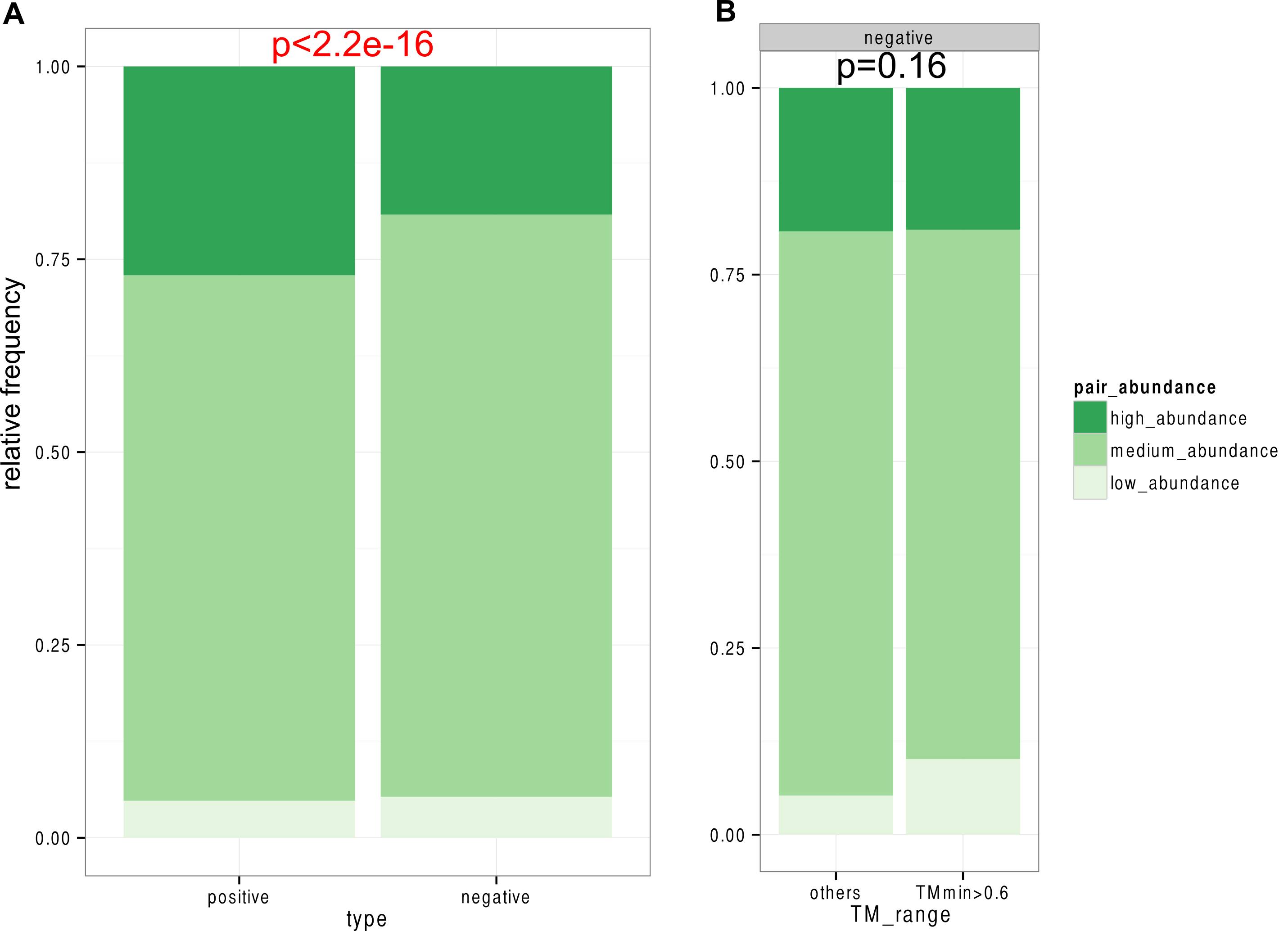


Figure S2. Analysis of protein abundance. The dataset is the same as the one used for the network analysis. A: abundance of proteins in positive *versus* negative pairs. The p-value of the Chi-squared test is shown. B: abundance of proteins in negative pairs with *versus* without structural precedents (TMmin>0.6). The p-value of the Fisher test is shown (counts were insufficient for a Chi-squared test).


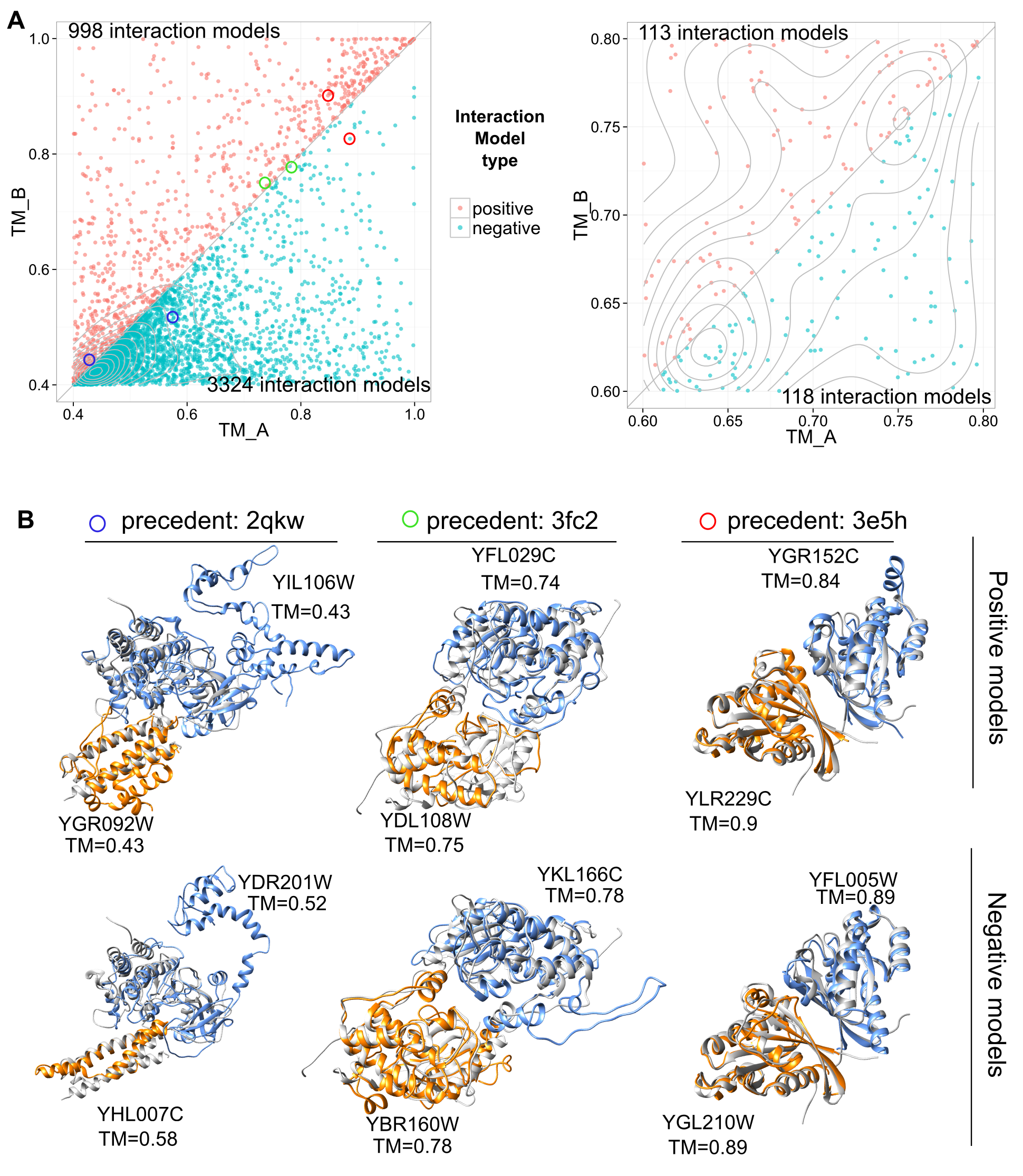


Figure S3. The structural space of positive and negative pairs. Positive interaction models are obtained from the union of the ‘positive BRS-direct’ data set and the ‘positive KUPS’ data set, and the negative interaction models are obtained from the union of the five negative data sets. A Left: global TM landscape of positive and negative pairs. Right: zoom on the TM-region in the 0.6-0.8 range. B: Examples of interaction models produced for positive and negative pairs with common structural precedents. At low scores (<0.6), the experimental complex 2qkw, a dimer between a plant kinase and a bacterial effector, supports a positive interaction between genes YGR092W (Mob1 kinase) and YIL106W (kinase regulator), but also a negative pair between YHL007C (kinase) and YDR201W (micro-tubule binding factor). At medium TM scores (range 0.6-0.8), the experimental complex 3fc2, a homo-dimer of human kinase, supports the interaction between YDL108W (kinase) and YFL029C (kinase activating kinase), and the negative pair between YBR160W (kinase) and YKL166C (kinase). At high TM scores (>0.8), the experimental complex 3e5h, a homo-dimer of human Ras-related protein, supports the interaction between YLR229C (GTPase) and YGR152C (Ras protein) and the negative pair between YGL210 (Rab GTPase) and YFL005W (Rab GTPase).


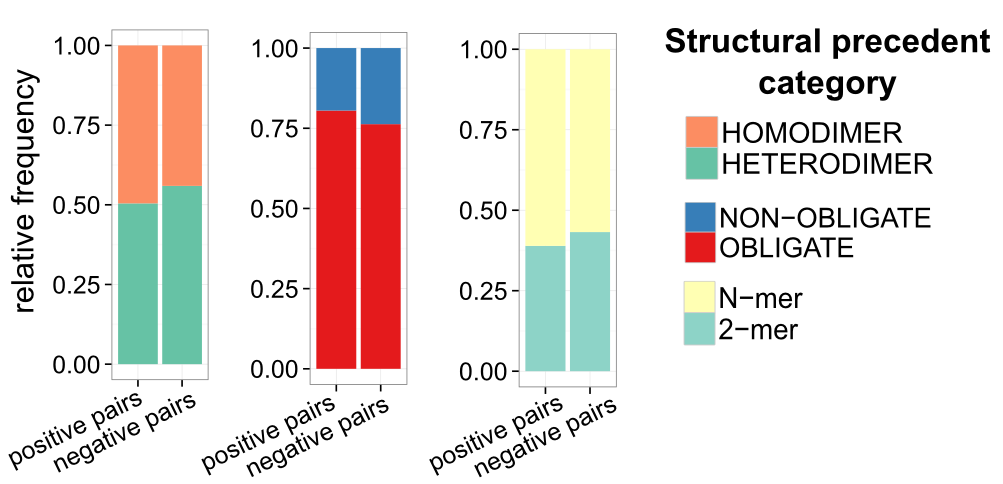


Figure S4. Characterization of the structural precedents of the interaction models in the TM range 0.6-0.8.


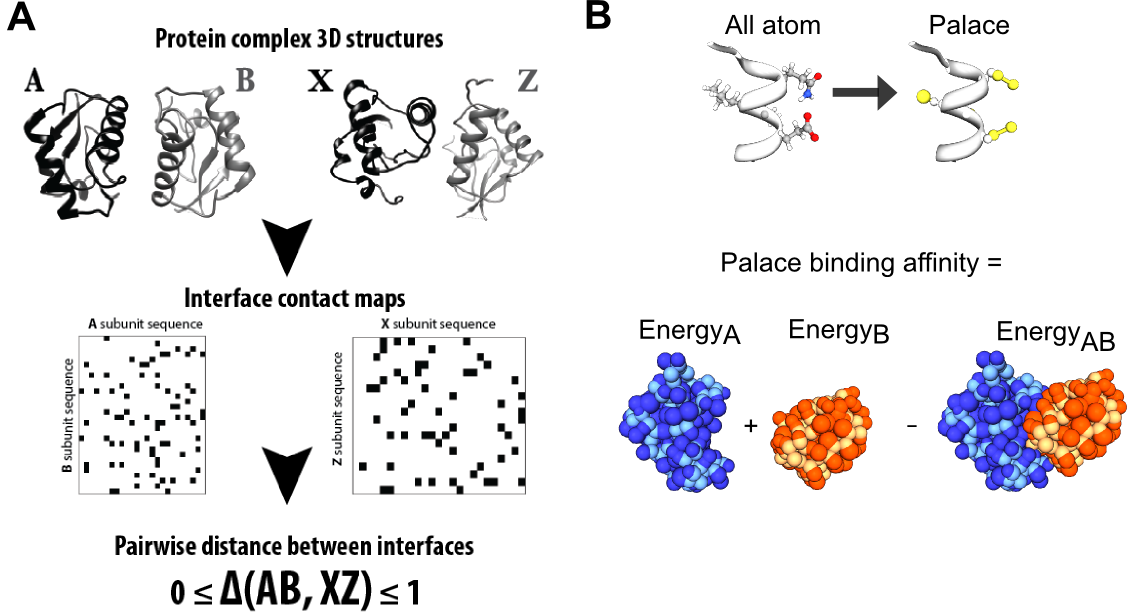


Figure S5. A: Illustration of the computation of distance between interface signatures. B: Illustration of the PaLaCe binding affinity prediction.


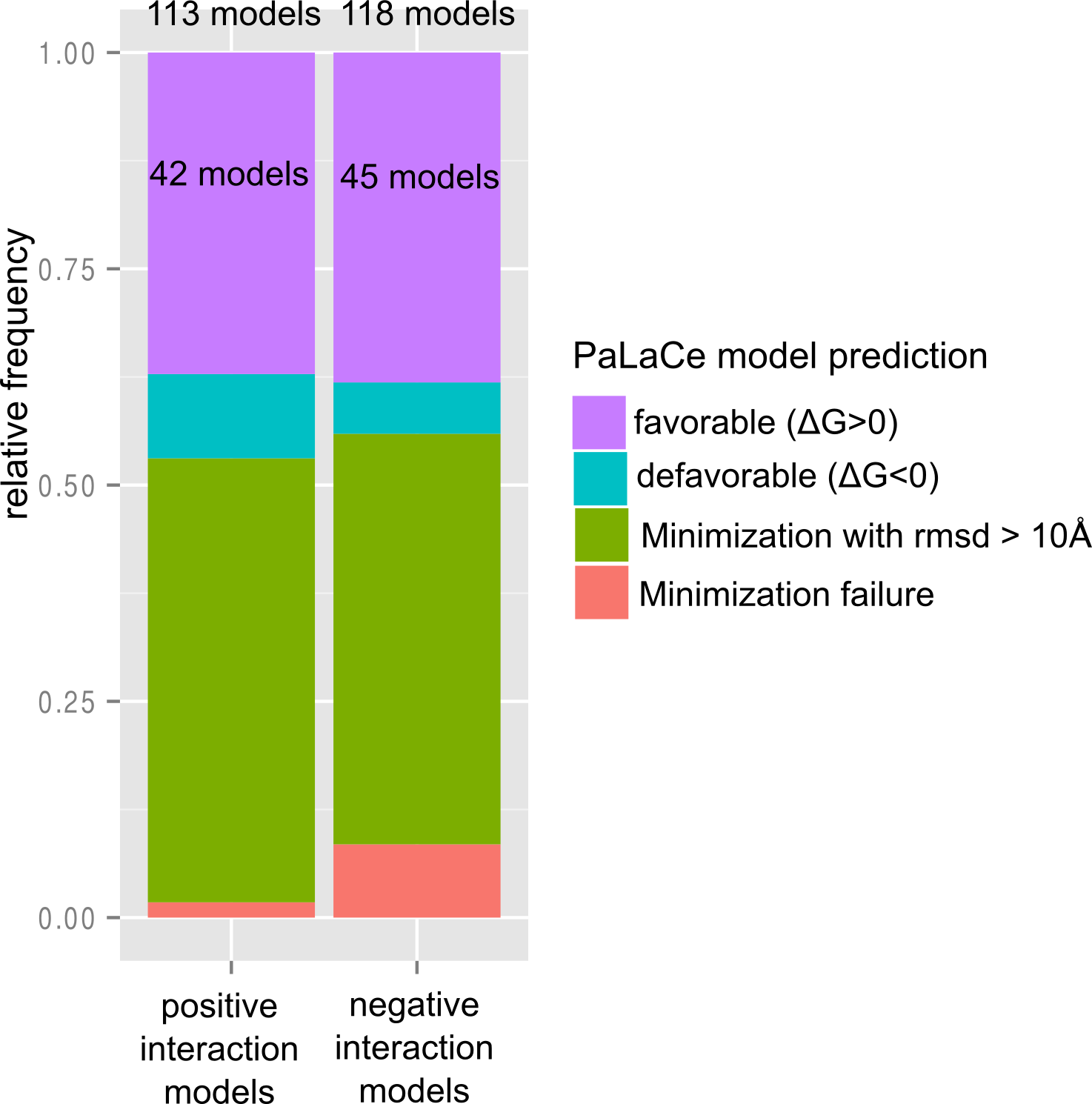


Figure S6. Binding affinity prediction by PaLaCe
